# Genomic structure, introgression, niche overlap and morphological variation challenge species delimitation in *Thymus* sect. *Mastichina*

**DOI:** 10.1093/aob/mcag151

**Published:** 2026-05-30

**Authors:** Diego Nieto-Lugilde, José Carlos del Valle, Esther María Martín-Carretié, David Doblas-Pruvost, Billy Williams-Marland, María Ángeles Ortiz, Francisco Javier Jiménez-López, Javier López-Tirado, Francisco José García-Cárdenas, Regina Berjano

**Affiliations:** Departamento de Botánica, Ecología y Fisiología Vegetal, Campus de Excelencia Internacional Agroalimentario CeiA3, Universidad de Córdoba, E-14071 Córdoba, Spain; Departamento de Biología Vegetal y Ecología, Universidad de Sevilla, E-41012 Sevilla, Spain; Departamento de Biología Vegetal y Ecología, Universidad de Sevilla, E-41012 Sevilla, Spain; Departamento de Biología Vegetal y Ecología, Universidad de Sevilla, E-41012 Sevilla, Spain; Departamento de Biología Vegetal y Ecología, Universidad de Sevilla, E-41012 Sevilla, Spain; Departamento de Biología Vegetal y Ecología, Universidad de Sevilla, E-41012 Sevilla, Spain; Departamento de Biología Vegetal y Ecología, Universidad de Sevilla, E-41012 Sevilla, Spain; Departamento de Botánica, Ecología y Fisiología Vegetal, Campus de Excelencia Internacional Agroalimentario CeiA3, Universidad de Córdoba, E-14071 Córdoba, Spain; Departamento de Biología Vegetal y Ecología, Universidad de Sevilla, E-41012 Sevilla, Spain; Departamento de Biología Vegetal y Ecología, Universidad de Sevilla, E-41012 Sevilla, Spain

**Keywords:** Cryptic species, ecological niche overlap, flower morphology, genomic, genetic introgression, morphological variation, species delimitation, *Thymus* sect. *Mastichina*

## Abstract

**Background and Aims:**

Delimitation of species remains a major challenge in understanding biodiversity, particularly in recently diverged lineages, where extensive morphological variability complicates taxonomic inference. In this study, we investigate patterns of morphological and ecological variation within *Thymus* sect. *Mastichina*, a Mediterranean lineage that includes the endangered species *Thymus albicans*. Previous genomic analyses revealed clear differentiation between two major groups corresponding to ploidy levels, in addition to additional genetic structure among diploid lineages.

**Methods:**

Here, we integrate this genomic framework with detailed analyses of floral morphology and ecological niches to assess the correspondence between phenotypic, ecological and genetic variation.

**Key Results:**

Our results reveal substantial overlap among genetic lineages at both morphological and ecological levels. Although some differentiation is associated with ploidy level, particularly in floral traits, extensive variability within several lineages obscures clear taxonomic boundaries based on discrete morphological characters. In contrast, some diploid lineages (notably the Cádiz and Algarve groups) show lower morphological variability but still both overlap in morphology and ecological niche. We morphologically characterized a recently discovered diploid lineage (Hercynian) that appears to be cryptic with the tetraploid *T. mastichina*. Patterns of admixture involving this Hercynian lineage, especially with the diploid Doñana group, contribute to pronounced phenotypic overlap, whereas no significant effect of tetraploid introgression on diploid morphology was detected. Ecological niche analyses indicate that niche differentiation is associated primarily with ploidy level rather than among diploid lineages, except for the Hercynian group.

**Conclusions:**

Overall, our results demonstrate a marked discordance between phenotypic variation and genetic structure, supporting the interpretation of *Thymus* sect. *Mastichina* as a complex of cryptic evolutionary lineages. These findings highlight the limitations of morphology-based taxonomy and emphasise the need for an integrative framework combining morphology, genome size and geographical distribution for taxa delimitation.

## INTRODUCTION

Recent advances in genetic analysis have transformed our understanding of species boundaries, often challenging classifications based solely on morphological criteria (Hending, 2025). Importantly, these approaches have revealed a dual pattern: they have exposed cases of taxonomic inflation, in which species boundaries were overestimated based on limited or potentially misleading phenotypic differences (Isaac *et al.*, 2004; Bickford *et al.*, 2007; Bateman and Rudall, 2023), while also uncovering substantial levels of cryptic diversity, identifying genetically distinct lineages with little or no morphological differentiation. This duality can have important implications for biodiversity assessment, because cryptic species might lead to misestimation of species richness and undermine the design and implementation of effective conservation strategies, especially for endemic or protected taxa (Bickford *et al.*, 2007; Poulin and Pérez-Ponce de León, 2017; Struck *et al.*, 2018). In this context, the contrasting evidence provided by genetic analyses has increasingly questioned the assumption that any observable phenotypic difference is sufficient to justify species status, underscoring the need for integrative approaches to species delimitation (Dayrat, 2005; Sukumaran and Knowles, 2017). Therefore, it is essential to determine whether cryptic lineages also exhibit meaningful differences in morphological traits, ecological niches or geographical distribution that are crucial for guiding field identification and conservation priorities (Bickford *et al.*, 2007; Hending, 2025).

The Mediterranean Basin stands out as a region where this question is especially relevant, owing to its unique combination of exceptional plant diversity, high levels of endemism and increasing ecological threats (Cuttelod *et al.*, 2008). Despite covering a small fraction of the surface of the Earth, the Mediterranean Basin hosts a remarkable diversity of habitats and supports high plant species richness, accounting for ∼10 % of global vascular plant diversity (Greuter, 1991; Myers *et al.*, 2000; Thompson, 2005; Blondel *et al.*, 2010; Nieto Feliner *et al.*, 2023). Notably, ∼60 % of plant species in this region are endemic, underscoring the crucial role of endemism in shaping its floristic diversity and originality (Greuter, 1991; Melendo *et al.*, 2003; Thompson *et al.*, 2005). This extraordinary botanical wealth is the result of complex abiotic influences, including climatic seasonal and spatial variability, soil heterogeneity and topographic variation, alongside a dynamic geoclimatic history shaped by tectonic activity, glaciations and shifts in sea levels (Holzapfel *et al.*, 1992; Matesanz and Valladares, 2014; Nieto Feliner *et al.*, 2023). In addition to these natural influences, anthropogenic pressures have also significantly impacted Mediterranean plant diversity (Médail and Diadema, 2006; Thompson, 2020; Peñuelas and Sardans, 2021). In this context, plant conservation emerges as a crucial priority for maintaining ecosystem stability, yet it faces major challenges, including limited assessments of species conservation status, insufficient funding and gaps in knowledge related to species distributions, ecology and/or reproductive biology (Havens *et al.*, 2014; Heywood, 2017).

The mint family (Lamiaceae Martinov) is a good example of plants adapted to the summer aridity that characterize the Mediterranean climate (Matesanz and Valladares, 2014; Aedo *et al.*, 2017), with thymes (*Thymus* L.) standing out for their remarkable diversification, in addition to other traits, such as aromaticity and frequent gynodioecy (Morales, 1986; Melendo *et al.*, 2003). However, this is a taxonomically complex group, because phenotypic plasticity, recent divergence and hybridization obscure diagnostic traits, blurring the boundaries between closely related taxa (Morales, 2002).

The genus *Thymus*, which includes mostly circum-Mediterranean species, is taxonomically divided into eight sections (Jalas, 1971; Morales, 1986). Among these, the section *Mastichina* Mill. comprises two species endemic to the Iberian Peninsula: *Thymus mastichina* L. and *Thymus albicans* Hoffmanns. & Link (Morales, 2010). *Thymus mastichina* is widely distributed across most of the Iberian Peninsula, whereas *T. albicans* has a much more restricted geographical range, being confined to the southwestern part of the Iberian Peninsula, mainly in Cádiz (Spain) and Algarve (Portugal). Indeed, *T. albicans* is listed as Critically Endangered (CR) in the Red List of Vascular Flora of Andalusia (Junta de Andalucía, 2012) and in the Red List of Spanish Vascular Flora (Moreno, 2008) and as Vulnerable (VU) in the IUCN Red List (Buira *et al.*, 2017). Taxonomic classifications have recognized two subspecies within *T. mastichina*: *T. mastichina* L. subsp. *mastichina*, a tetraploid taxon widely distributed throughout most of the Iberian Peninsula, and *T. mastichina* subsp. *donyanae* R.Morales, a diploid taxon mainly inhabiting the sandy soils of Doñana National Park (Huelva, Spain). However, recent phylogenomic studies have revealed that *Thymus* sect. *Mastichina* is more accurately divided into two well-supported clusters: one comprising tetraploid individuals, mostly with a distribution corresponding to *T. mastichina* subsp. *mastichina sensu*  Morales (2010), and the other consisting of diploid individuals, taxonomically classified by Morales (2010) as *T. albicans* and *T. mastichina* subsp. *donyanae*, but also some populations identified as *T. mastichina* subsp. *mastichina* (García-Cárdenas *et al.*, 2026). According to that study, the diploid cluster can be divided further into four genetic groups, each exhibiting a clear geographical pattern: Algarve (Algarve, Portugal), Doñana (Huelva, Spain), Cádiz (Cádiz, Spain) and Hercynian (Córdoba, Spain; and Serra da Estrela, Portugal) (García-Cárdenas *et al.*, 2026). Moreover, in most populations signs of genetic introgression (i.e. the incorporation of genetic material from one species or population into another) have been detected, at both intraploidy level (among diploid genetic groups) and interploidy level (between diploids and tetraploids; García-Cárdenas *et al.*, 2026).

By introducing new alleles and genetic combinations, introgression might increase phenotypic variation, altering characteristics such as size, shape, structure or coloration of vegetative and reproductive organs (Aguillon *et al.*, 2022). This enhanced variability can lead to the emergence of novel traits or modifications of existing ones, potentially affecting ecological adaptation and population dynamics (Suarez-Gonzalez *et al.*, 2018; Villa-Machío *et al.*, 2024). On the contrary, introgression can also blur or modify species-specific morphological features, complicating taxonomic identification and impacting local evolutionary trajectories (Thórsson *et al.*, 2001).

Given the cryptic genetic diversity, its mismatch with previous taxonomical works (Morales, 2010) and the prevalence of introgression in *Thymus* sect. *Mastichina* (García-Cárdenas *et al.*, 2026), it seems necessary to analyse the morphological and ecological variation within the group. Here, we aim to analyse morphological and ecological data in *Thymus* sect. *Mastichina* to discern patterns among ploidy levels and/or genetic clusters. More specifically, we aim to distinguish whether there are recognizable morphological or ecological differences between the different lineages (ploidy levels and genetic groups) that could contribute to clarify the taxonomy of the section and to assess the role of introgressions in shaping phenotypic traits. We hypothesise that: (1) the tetraploid individuals display a wider phenotypical and ecological variability than the diploid individuals; (2) the different genetic groups might display some different but overlapping morphologies and/or ecologies; and (3) introgression of the tetraploid genome into diploid populations might affect morphological and ecological features in diploids, causing overlap in features of the species.

## MATERIALS AND METHODS

To test our hypotheses, we studied multiple populations of *Thymus* sect. *Mastichina* across the Iberian Peninsula. For each population, we characterized the main morphological traits of their representatives (namely floral traits commonly used in the taxonomy of the genus), their abiotic environment through soil and climate conditions, and their biotic environment through vegetation quadrats. Finally, we complemented our dataset by gathering existing information on the ploidy level and genetic group assignment of each population. These morphological and environmental datasets were evaluated using a suite of univariate and multivariate statistical approaches to test for differentiation among ploidy levels and genetic clusters. Finally, we also examined the influence of ancestry introgression on floral trait variation.

### Plant material

**Fig. 1.**
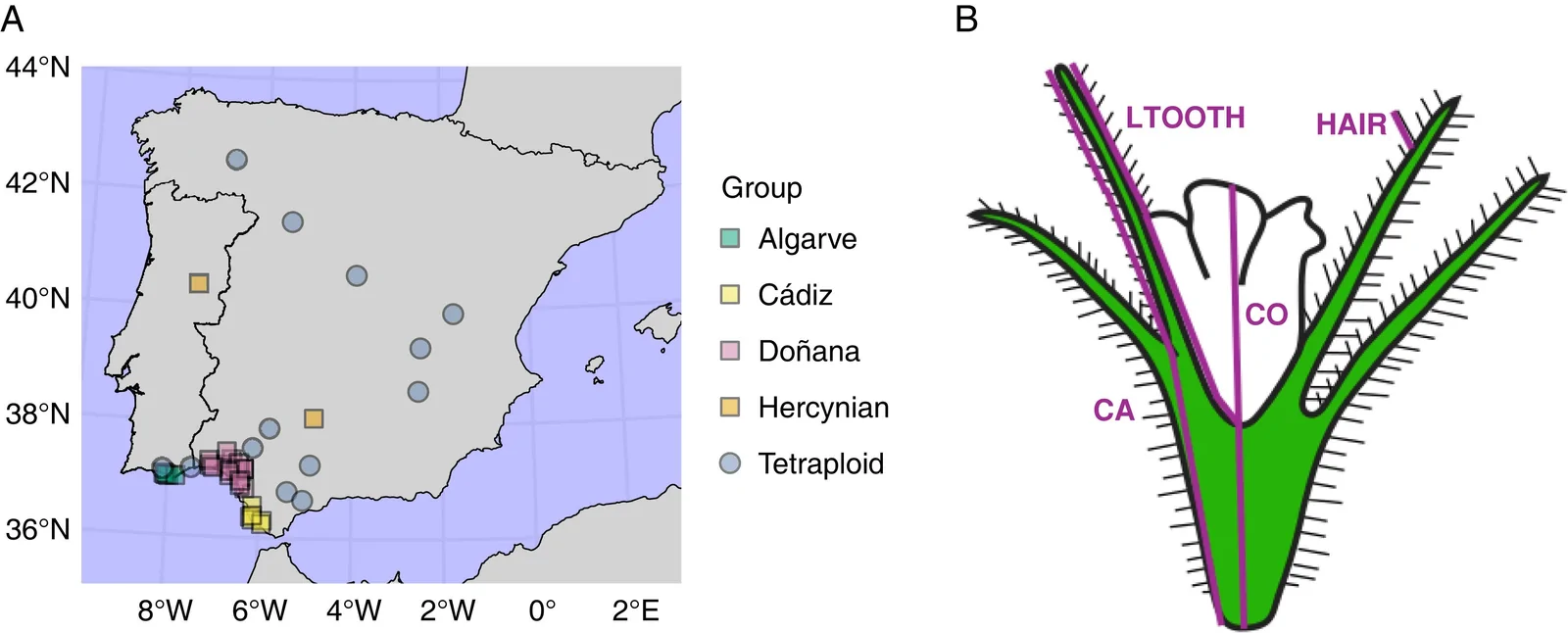
Geographical sampling of populations from *Thymus* sect. *Mastichina* and schematic representation of measured floral traits. (A) The map depicts the Iberian Peninsula, showing sampling locations coloured by genetic group (García-Cárdenas *et al.*, 2026). (B) The diagram represents a flower, indicating the morphological traits measured: corolla length (CO), calyx length (CA), length of the longest calyx tooth (LTOOTH) and length of the longest calyx hair (HAIR). The studied populations are colour-coded according to the five genetic groups identified by García-Cárdenas *et al.* (2026): Tetraploid, Hercynian, Algarve, Doñana and Cádiz. Circles and squares represent diploid and tetraploid locations, respectively. The map is in UTM projection for the zone 30N and datum WGS84.

We collected samples from 39 populations spanning the Iberian Peninsula, encompassing the distribution of *Thymus* sect. *Mastichina*. Given that the distribution area of *T. albicans* is restricted to the southwestern region of the Iberian Peninsula, sampling effort was concentrated in this area (Fig. 1; Supplementary Data Table S1). From May to June 2023, during peak flowering, we collected samples from 15 hermaphroditic and 15 female plants per population. Individuals were randomly selected in the population at approximately the same phenological stage. Marked individuals were at a minimum distance of 5 m between them to reduce the probability of sampling siblings. At least one flowering stem per individual was collected and preserved as a herbarium specimen. One herbarium voucher per population was deposited in the SEV herbarium of the University of Seville (Supplementary Data Table S1).

### Genomic data and ploidy level

The studied populations were the same as those used in the genomic analysis described by García-Cárdenas *et al.* (2026); consequently, we used the information from that study to obtain variables that characterize the studied individuals. Specifically, García-Cárdenas *et al.* (2026) estimated 2C DNA content of the same individuals of this study by flow cytometry, allowing the classification of individuals into diploids and tetraploids. Additionally, genetic clustering and ancestry estimates derived from genome-wide single nucleotide polymorphism data generated through HybSeq further separated the Tetraploid group and four diploid genetic groups: (1) Algarve; (2) Doñana; (3) Cádiz; and (4) Hercynian. Lastly, we obtained the percentage of genetic ancestry for all individuals also from ADMIXTURE analysis. With all this information, we created two categorical variables (i.e. ploidy level and genetic group) and one continuous variable (i.e. percentage of genetic ancestry) for each plant individual.

### Morphological analysis

For the morphological characterization of individuals from the studied populations, we measured the following floral traits (Fig. 1; Supplementary Data Table S2): calyx length (CA), corolla length (CO), length of the longest calyx tooth (LTOOTH), length of the longest hair on calyx tooth (HAIR), number of flowers in the inflorescence (FL_INFL) and diameter of the inflorescence (D_INFL). Additionally, we calculated the relative size (PROP_LTOOTH) of the longest calyx tooth (LTOOTH) compared with the calyx length (CA). Floral traits were measured in one to seven open flowers from 3–12 collected individuals in each population (including both hermaphroditic and female individuals), yielding a total of 965 flowers. These flowers were extracted from their inflorescences and digitized with an Epson Expression 11000XL high-resolution scanner. The resulting images were analysed using the software ImageJ v.1.53t (Schneider *et al.*, 2012).

Additionally, we conducted a preliminary analysis of microscopic features (i.e. pollen and stomata sizes). More specifically, we measured the minor and major pollen axes (D_MIN and D_MAX, respectively) and the length and width of the stomata (STOMA_L and STOMA_W, respectively). For pollen, ten grains per individual were analysed from one or two individuals across seven populations (*n* = 100). For stomata, ten measurements per individual were taken from three individuals in seven populations (*n* = 210). Samples were prepared following the protocol described by Scarpeci *et al.* (2017) and using a Leica DFC-490 microscope. However, the preliminary results suggested that there were no different signals in the microscopic features, hence we did not continue to measure the rest of the populations fully (Supplementary Data Fig. S1; Tables S2 and S3).

### Environmental characterization of natural populations

To characterize abiotic environmental conditions in each population, we gathered climatic data and soil samples. Climate was characterized by the extended bioclimatic (Bioclim+) variables from the Chelsa Climate Database v.2.1 (Brun *et al.*, 2022). We extracted values for aridity index (AI), the bioclim variables (BIO1–19), mean climate moisture index (CMI_MEAN), growing degree days heat sum at >5 °C (GDD5) and mean near-surface wind speed (SFCWIND_MEAN) using the geographical coordinates of the populations recorded in the field when collecting plant samples. To reduce the multidimensionality, we performed a principal component analysis (PCA) on the whole set of variables and retained the first five axes for further analysis.

We obtained soil variables for each studied population from a prior analysis of three soil samples from the same localities, as published by del Valle *et al.* (2026). Soil variables included pH, electrical conductivity (EC), water retention potential (WRP), water retention capacity (WRC) and organic matter (OM).

To characterize the plant community, at each population we set three quadrats of 4 m^2^ following Lavergne *et al.* (2004). Vascular plants were inventoried by means of point contact for 100 points for each quadrat (20 cm × 20 cm grid). Coverage of plant species was assessed by summing the contact points, then dividing by 100 (total number of contact points). Additionally, we completed the list of companion species by randomly walking around the quadrats (15–20 m diameters) and identifying other species absent in the quadrats. In some sites, the companion species were not represented in the quadrats but might be important in the community (e.g. tree or shrub species), hence they were also included in the inventories with the lowest coverage (i.e. 1 %).

### Statistical analyses

To explore overall patterns of variation in morphological traits and environmental variables (both bioclimatic and soil), we assessed differences between ploidy levels (diploids vs. tetraploids) and among the five genetic clusters identified within *Thymus* sect. *Mastichina*. We also explored morphological differences between sexes (hermaphrodites and female individuals). Given that the data did not meet normality assumptions, we used non-parametric tests (Mann–Whitney *U*-test for comparison between ploidy levels and Kruskal–Wallis tests, followed by Dunn’s *post hoc* test, for comparison between genetic groups). Additionally, to evaluate potential differences between lineages in multivariate spaces, for both morphological and environmental variables, we performed a PCA using functions from base R and retained the first two axes. Then, we visualized the PCA results using the *factoextra* v.1.0.7 R package (Kassambara and Mundt, 2020). Finally, we analysed variation in calyx size (one of the main features used in taxonomy of the section) using Bayesian hierarchical linear models fitted using the *brms* v.2.23.0 R package (Bürkner, 2021), partitioning phenotypic variance among genetic groups, populations, individuals and residual error. Models were fitted using Hamiltonian Monte Carlo sampling, and posterior distributions of variance components and their proportional contributions to total variance were summarized using posterior means and 95 % credible intervals. Separate hierarchical models were fitted for each genetic group to characterize and compare the internal distribution of variance among populations, individuals and residual components within groups.

To study differences in the ecological communities, we used principal coordinates analysis (PCoA), by standardizing the species cover data with the Hellinger transformation and calculating the Bray–Curtis distance between quadrats, as applied in the decostand and vegdist functions in the *vegan* v.2.7.2 R package (Oksanen *et al.*, 2018).

An *ad hoc* analysis of morphological differentiation among genetic groups was performed using Gaussian mixture models within the *mclust* v.6.1.2 R package. After imputation of missing values, model-based clustering was used to characterize the multivariate structure of morphological traits, with model selection based on the Bayesian information criterion. A series of alternative grouping hypotheses was defined *a priori* and evaluated using model-based discriminant analysis (MclustDA), and their relative support was assessed using Bayes factors under uniform priors (Supplementary Data Table S4). Additionally, niche overlap among clades was quantified in reduced environmental spaces derived from PCA of bioclimatic and soil variables and from PCoA of ecological communities, using a Bayesian framework implemented in the *nicheROVER* v.1.1.2 R package to estimate probabilistic niche regions and their pairwise overlap. Uncertainty in overlap estimates was quantified using 95 % credible intervals derived from the posterior distributions (Supplementary Data Table S5).

Finally, to test how ancestry introgression among genetic groups affects floral morphology, we used the genomic data generated by García-Cárdenas *et al.* (2026). More specifically, we fitted regression models, using the percentage of ancestry to different genetic groups as predictors and each floral trait as a response variable. Within each of the five main genetic groups, we modelled the effect of ancestry from the remaining genetic groups, which we interpret as ancestry introgression, on trait variation. Initially, we fitted Gaussian linear mixed models with a random intercept for sex (i.e. females and hermaphrodites) with the *glmmTMB* v.1.1.13 R package (McGillycuddy *et al.*, 2025) and compared them with models without the random effect using the corrected Akaike information criterion (AICc); when the random effect did not improve model fit (ΔAICc < 2), we retained the simpler linear models. In addition to multivariate models including several ancestry components simultaneously, we also fitted univariate models with a single percentage of genetic ancestry as predictor, to assess the effect of each ancestry component independently. Model assumptions were checked by visual inspection of residuals Q–Q plot.

## RESULTS

### Floral morphology

**Fig. 2.**
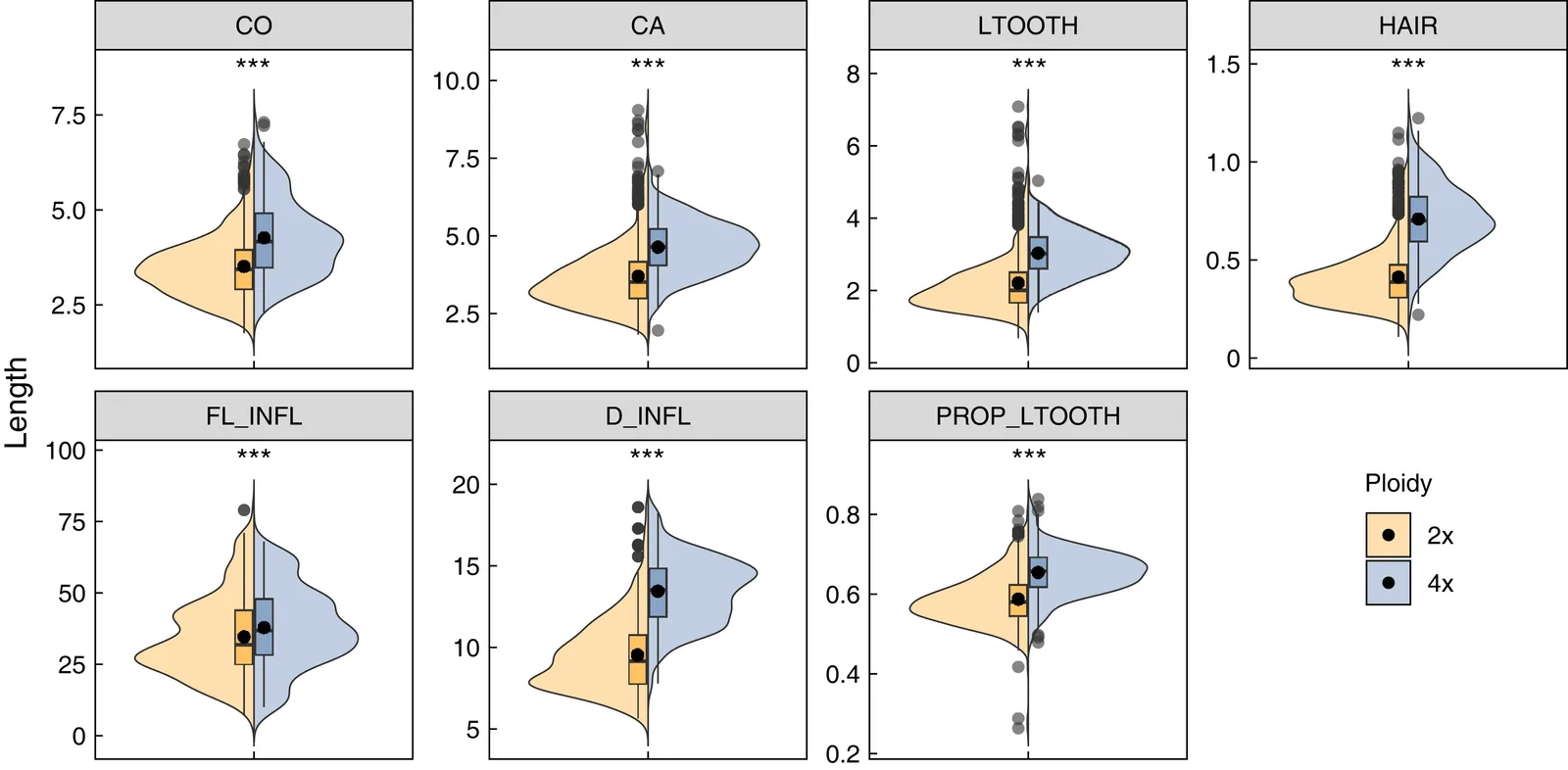
Comparison of morphological traits between ploidy levels. Violin plots combined with boxplots show the full distribution of values obtained for each measured morphological trait: corolla length (CO), calyx length (CA), length of the longest calyx tooth (LTOOTH), length of the longest calyx hair (HAIR), number of flowers in the inflorescence (FL_INFL), inflorescence diameter (D_INFL) and proportion of the calyx tooth (PROP_LTOOTH). All metrics are in millimetres, except for FL_INFL, which is expressed as the number of flowers, and PROP_LTOOTH, which is dimensionless. Boxplots represent the interquartile range (IQR); the thin black line indicates 1.5× the IQR, the central line shows the median, and the black dot represents the mean sample value; dark grey dots outside the IQR represent the outlier values. Asterisks denote significant differences between ploidy levels (***P < 0.001).

When comparing diploid and tetraploid individuals, we found that the tetraploids exhibited significantly larger values of floral traits than diploids across all measured parameters (Fig. 2; Supplementary Data Tables S2 and S3), except for microscopic features (pollen grain and stomata size; Supplementary Data Fig. S1). Despite these significant differences, we discovered substantial variability and overlapping between the two ploidy levels (Fig. 2). These results were corroborated by the overlapping position of the clouds of points in the PCA (Supplementary Data Fig. S2), which did not clearly separate some tetraploid and diploid individuals. The first two components of the PCA accounted for 69.2 and 12.4 % of the total variation, respectively.

**Fig. 3.**
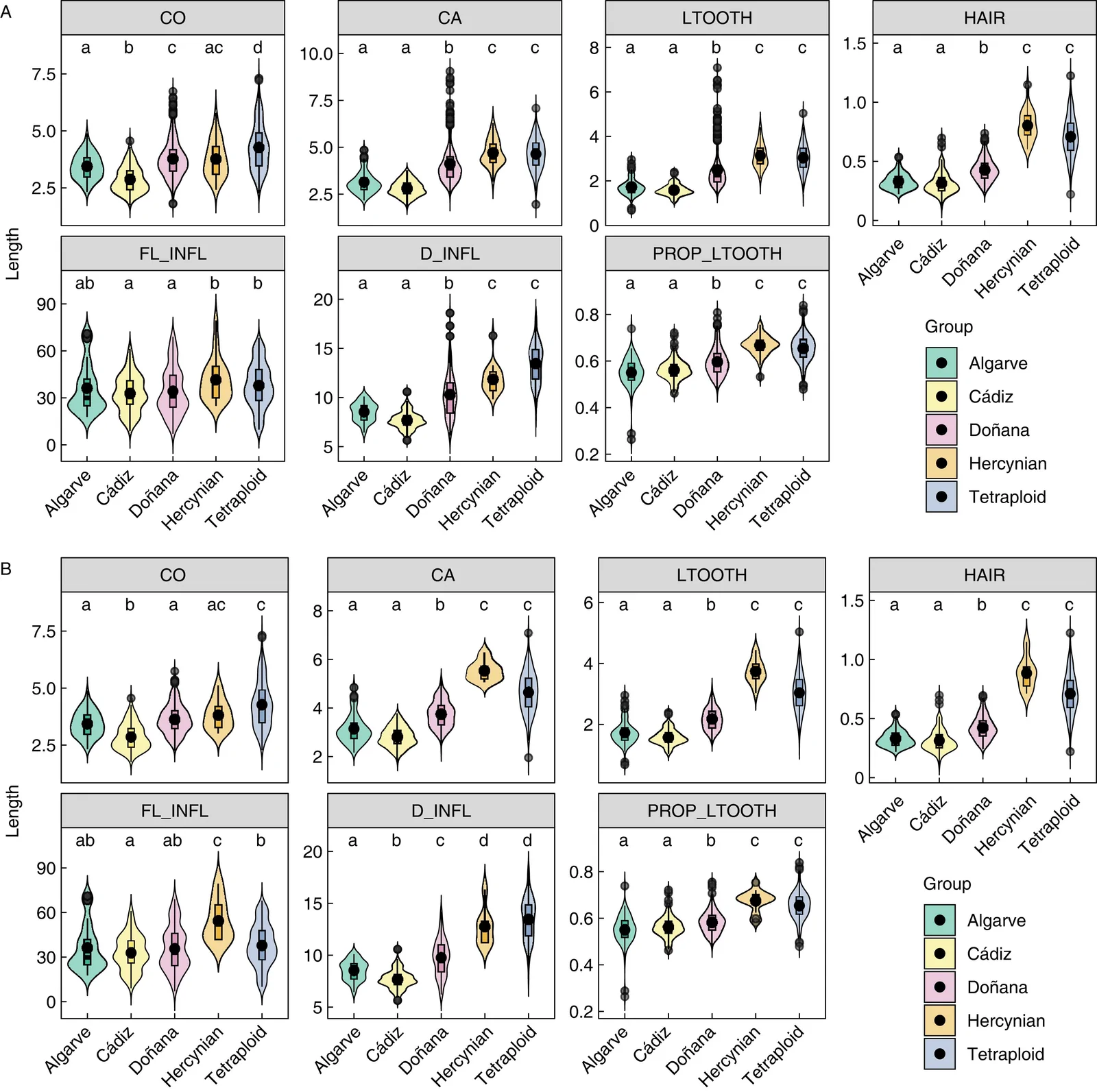
Comparison of morphological traits across genetic groups with (A) and without (B) individuals from populations with mean self-ancestry values of <75 %. Violin plots combined with boxplots show the full distribution of values obtained for each measured morphological trait: corolla length (CO), calyx length (CA), length of the longest calyx tooth (LTOOTH), length of the longest calyx hair (HAIR), number of flowers in the inflorescence (FL_INFL), inflorescence diameter (D_INFL) and proportion of the calyx tooth (PROP_LTOOTH). All metrics are in millimetres, except for FL_INFL, which is expressed as the number of flowers, and PROP_LTOOTH, which is dimensionless. Boxplots represent the interquartile range (IQR); the thin black line indicates 1.5× the IQR, the central line shows the median, and the black dot represents the mean sample value; dark grey dots outside the IQR represent the outlier values. Different letters above violin plots represent significant pairwise differences among genetic groups according to *post hoc* tests on Dunn’s test (adjusted *P* < 0.05).

In general, 41 % of the observed variance for CA was attributed to differences among the five genetic groups, 40 % was attributed to differences among populations and only 14 % within individuals (Table 1). When considering the five genetic groups, we observed that all individuals from Algarve and Cádiz genetic groups exhibited lower values of floral traits (e.g. CA is always <4 mm, except for population D04 from Algarve; Supplementary Data Table S3). In contrast, individuals of the Tetraploid group tended to exhibit significantly larger values of floral traits (Fig. 3; Supplementary Data Table S2). However, it is important to note that small values of floral traits (e.g. CA < 4 mm) were also observed in most populations of the Tetraploid group. In some tetraploid populations (e.g. M13), mean values of floral traits were even comparable to those of diploid populations from the Cádiz and Algarve groups (Supplementary Data Table S2).

**Table 1.** Bayesian hierarchical variance partitioning of calyx size across genetic groups. For the full dataset (‘All groups’), variance (var.) components for the genetic group, population, individual and residual levels are shown, as estimated from a hierarchical model fitted with the brms R package. For each genetic group analysed separately (Algarve, Cádiz, Doñana, Hercynian and Tetraploid), variance components correspond to population, individual and residual levels. Values represent posterior means, with 95 % credible intervals in parentheses. Total variance is the sum of all estimated components within each model. Proportional (prop.) contributions indicate the fraction of total variance attributable to each hierarchical level.

Genetic group	Genetic group var.	Population var.	Individual var.	Residual var.	Total var.	Genetic group prop.	Population prop.	Individual prop.	Residual prop.
All groups	1.13 (0.07–4.89)	0.80 (0.46–1.34)	0.26 (0.21–0.33)	0.11 (0.10–0.12)	2.30 (1.16–6.11)	0.41 (0.05–0.81)	0.40 (0.12–0.70)	0.14 (0.05–0.24)	0.06 (0.02–0.10)
Algarve	–	0.55 (0.07–2.40)	0.14 (0.07–0.26)	0.07 (0.05–0.10)	0.76 (0.26–2.63)	–	0.61 (0.23–0.92)	0.26 (0.05–0.56)	0.14 (0.03–0.30)
Cádiz	–	0.04 (0.00–0.19)	0.12 (0.07–0.20)	0.05 (0.04–0.07)	0.21 (0.13–0.37)	–	0.14 (0.00–0.54)	0.61 (0.31–0.79)	0.26 (0.13–0.40)
Doñana	–	1.62 (0.74–3.31)	0.21 (0.15–0.30)	0.06 (0.05–0.08)	1.90 (1.02–3.59)	–	0.84 (0.72–0.93)	0.13 (0.06–0.22)	0.04 (0.02–0.06)
Hercynian	–	1.93 (0.11–9.81)	0.21 (0.07–0.55)	0.05 (0.03–0.08)	2.18 (0.33–10.0)	–	0.76 (0.28–0.98)	0.19 (0.02–0.63)	0.05 (0.00–0.15)
Tetraploid	–	0.26 (0.06–0.72)	0.42 (0.28–0.60)	0.20 (0.16–0.24)	0.88 (0.63–1.34)	–	0.28 (0.09–0.55)	0.49 (0.29–0.66)	0.23 (0.14–0.32)

Interestingly, the diploid Hercynian group also tended to exhibit significantly larger values of floral traits, comparable to those of the Tetraploid group (Fig. 3; Supplementary Data Table S2), and individuals from populations of the Doñana genetic group display the highest variability in most of the floral traits (i.e. CO, CA, LTOOTH and D_INFL) and a broad overlap with all other groups, with some populations with individuals similar in size to the smaller-flowered Algarve and Cádiz groups, others with intermediate characters (e.g. CA ranging between 4 and 5 mm) and still others with large values of flower traits resembling those of the Tetraploid and Hercynian genetic groups.

One population of western range within the Doñana group (M03) exhibited the largest calyx sizes among all populations (Supplementary Data Table S3). In fact, the Doñana group was the one that exhibited a higher percentage of variance attributable to differences among populations (85 %; Table 1). In contrast, populations from Cádiz genetic group showed a lower percentage of variance attributable to differences among populations (14 %), and most of variance (61 %) in this group was within individuals; still, variance within Cádiz individuals was the lowest observed in any genetic group (mean = 0.12; Table 1).

In line with these morphological trends, the PCA clearly separated the Tetraploid and Hercynian clusters from the diploid Algarve and Cádiz genetic groups. However, individuals from Doñana showed a broad overlap with all other groups, further illustrating the high degree of morphological variability across genetic groups (Supplementary Data Fig. S3). The model-based clustering and discriminant analyses strongly supported the presence of three morphological groups and largely corroborated the patterns observed in both the PCA and univariate analyses (Supplementary Data Table S4), highlighting a clear differentiation of Tetraploid and Hercynian individuals from the intermediate flower size in Doñana and the smaller flower in Algarve and Cádiz groups.

### Soil variables

**Fig. 4.**
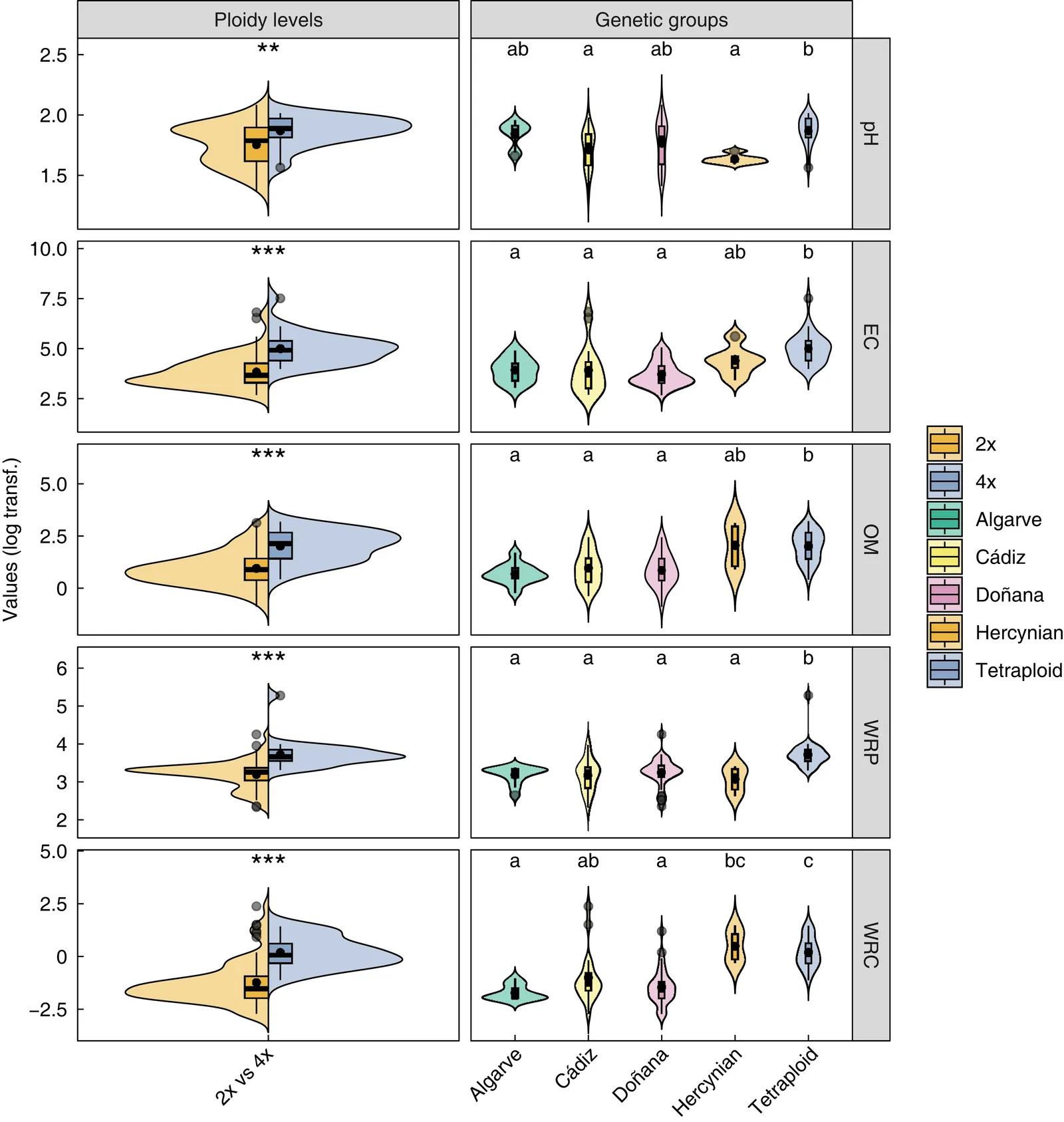
Comparison of soil characteristics across ploidy levels and genetic groups. Violin plots show the distribution of values for diploid (2*x*) and tetraploid (4*x*) cytotypes (left column) and for the main genetic groups (right column: Algarve, Cádiz, Doñana, Hercynian and Tetraploid). Each panel corresponds to a different trait: electrical conductivity (EC), content in organic matter (OM), pH, water retention potential (WRP) and water retention capacity (WRC). Boxplots represent the interquartile range (IQR); the thin black line indicates 1.5× the IQR, the horizontal central line shows the median, and the black dot within the boxplot represents the mean; dark grey dots outside the IQR represent the outlier values. Asterisks denote significant differences between ploidy levels (**P < 0.01 and ***P < 0.001). Different letters above violin plots represent significant pairwise differences among genetic groups according to *post hoc* tests on Dunn’s test (adjusted *P* < 0.05).

The tetraploid and diploid populations differed significantly in all measured soil variables, with higher pH, EC, OM, WRP and WRC values in tetraploids; however, substantial overlap in value ranges was observed (Fig. 4). When analysing soil variables by genetic groups, significant differences among diploids (Algarve, Cádiz, Doñana and Hercynian) were observed only for WRC, with Hercynian populations generally exhibiting higher values, comparable to those of tetraploids. This pattern was also reflected in EC and OM, where Hercynian populations showed values similar to tetraploid populations. In contrast, WRP was the only variable for which the Hercynian group did not differ from the other diploids but differed significantly from tetraploids. For pH, Algarve and Doñana exhibited values more similar to tetraploids than Cádiz and Hercynian.

For pH, variability was highest in the Algarve and Doñana groups and in tetraploid populations, whereas the Hercynian group showed the lowest values. A considerable overlap among both ploidy levels and genetic groups was evident in the PCA (Supplementary Data Fig. S4). Soil EC, OM and WRP aligned with axis 1 of the PCA, pH aligned with axis 2, and WRC aligned between the two axes. The first two components of the PCA represented 56.4 and 21.2 % of the total variation, respectively. The populations displayed in the biplot of these two axes showed consistent overlap among ploidy levels and genetic groups. The niche overlap estimates were broadly consistent with the PCA results, confirming extensive overlap among diploid genetic groups, particularly between Algarve, Cádiz and Doñana, with high and often symmetrical overlap values (Supplementary Data Table S5). In addition, the Hercynian group showed substantial overlap with Cádiz and Doñana, supporting its intermediate position observed in the multivariate analyses. In contrast, overlap involving the Tetraploid clade was generally lower and strongly asymmetric, indicating partial differentiation despite the broad overlap in ordination space.

### Bioclimatic variables

**Fig. 5.**
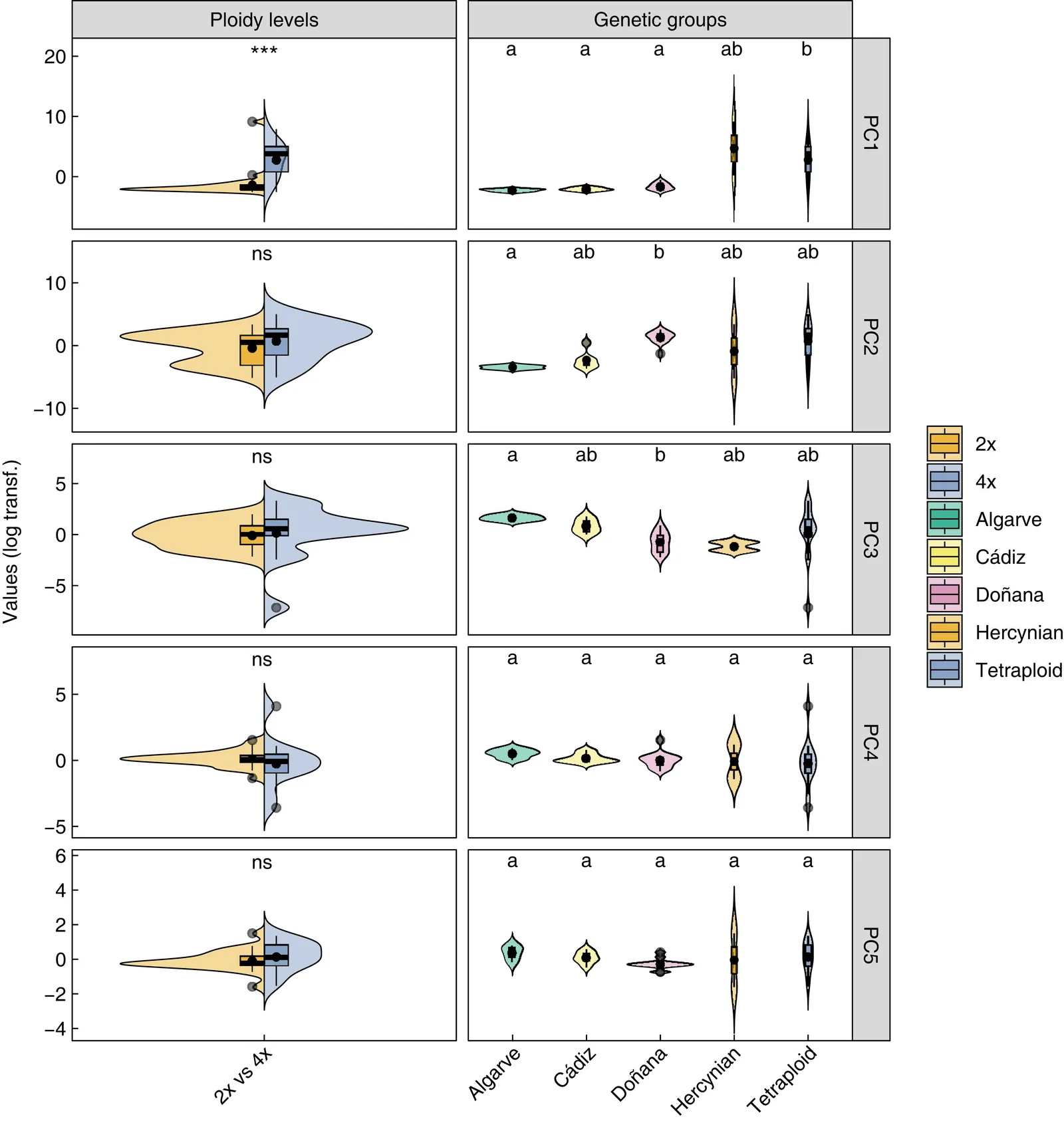
Comparison of bioclimatic variables across ploidy levels and genetic groups. Violin plots show the distribution of values for diploid (2*x*) and tetraploid (4*x*) cytotypes (left column) and for the main genetic groups (right column: Algarve, Cádiz, Doñana, Hercynian and Tetraploid). Each panel corresponds to a different principal component (i.e. from PC1 to PC5). Boxplots represent the interquartile range (IQR); the thin black line indicates 1.5× the IQR, the horizontal central line shows the median, and the black dot within the boxplot represents the mean; dark grey dots outside the IQR represent the outlier values. Asterisks denote significant differences between ploidy levels (***P < 0.001; ns, non-significant). Different letters above violin plots represent significant pairwise differences among genetic groups according to *post hoc* tests on Dunn’s test (adjusted *P* < 0.05).

When analysing bioclimatic variables through the first five axes of a PCA (Fig. 5), those axes represented 37.1, 26.9, 12.2, 10.0 and 4.1 % of the variation, respectively. We found significant differences between ploidy levels for axis 1 of the PCA (PC1), although some overlap was observed, mainly attributable to the Hercynian diploid populations and the high variability within the Tetraploid group (Supplementary Data Fig. S5). Furthermore, when analysing the bioclimatic variables across the genetic groups, we found no PCA axis showing significant differences, except for Tetraploid on axis 1 (PC1) and between Algarve and Doñana on axes 2 and 3 (PC2 and PC3) (Fig. 5). The biplot of the populations in the two first axes of the PCA shows these overlaps more clearly. Populations spread through the first axis of the PCA, with the genetic groups Algarve, Cádiz and Doñana clustering in the left of the biplot, whereas the two populations of the Hercynian group spread towards the right. The Tetraploid group overlaps extensively with Doñana and Hercynian populations (Supplementary Data Fig. S5). The niche overlap estimates largely corroborated the PCA results (Supplementary Data Table S5), highlighting substantial overlap of the Doñana and Hercynian groups within the Tetraploid niche, and of the Algarve group within the Cádiz niche. Most other comparisons showed low overlap values, wide credible intervals and/or pronounced asymmetry.

### Ecological communities

**Fig. 6.**
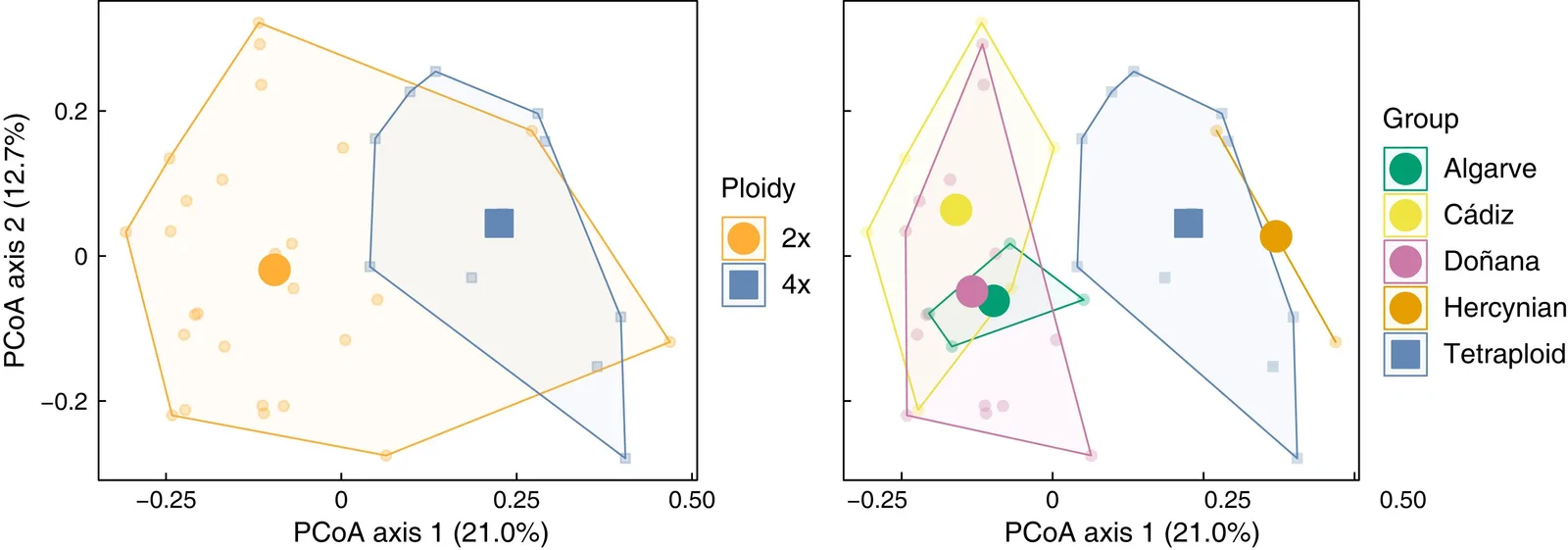
Principal coordinates analysis (PCoA) of vegetation dissimilarity across ploidy levels and genetic groups for populations of *Thymus* sect. *Mastichina*. PCoA ordination of samples coloured by ploidy level [diploid (2*x*) or tetraploid (4*x*); left plot] and genetic groups (Algarve, Cádiz, Doñana, Hercynian and Tetraploid; right plot). Convex hulls and centroids summarise the multivariate dispersion and central tendency for each group. Circles and squares represent diploid and tetraploid populations, respectively.

The composition of the plant communities associated with the studied populations showed no clear differentiation across ploidy levels, as revealed by the PCoA based on Hellinger-transformed species cover. The first two PCoA axes explained 21.0 and 12.7 % of the variation in community composition, respectively. Jointly, both axes did not separate diploid and tetraploid populations (Fig. 6), with complete overlap between the two ploidy levels. When examined at the level of genetic groups, Algarve, Cádiz and Doñana populations formed overlapping but distinguishable clusters, whereas the Hercynian and Tetraploid genetic groups were clearly isolated from the remaining groups, indicating greater compositional distinctiveness (Fig. 6). Directional niche overlap analyses based on plant community composition were consistent with the ordination results (Supplementary Data Table S5), showing generally high overlap among Algarve, Cádiz and Doñana populations and lower overlap involving the Hercynian group. In contrast, Tetraploid and Hercynian populations showed relatively high overlap values, supporting the compositional similarity and isolation of these two groups observed in the ordination space.

Hercynian populations exhibited the lowest species richness in their communities (eight species), whereas the rest of the groups showed much higher richness, varying from 29 to 39 species (mean = 35.25). *Pistacia lentiscus* L. was the only species that appeared in communities of the five genetic groups. No single species occurred exclusively within the communities of any specific *Thymus* genetic group. Multiple species appeared in communities of both Tetraploid and most diploid genetic groups [e.g. *Lavandula pedunculata* (Mill.) Cav., missing only from Cádiz group; *Pinus pinea* L., *Chamaerops humilis* L., *Rhamnus alaternus* L. or *Cistus salviifolius* L. missing only from Hercynian group]. However, communities of the southwestern diploid genetic groups (Algarve, Doñana and Cádiz) shared some characteristic species (e.g. *Cistus calycinus* L. or *Cistus halimifolius* L.).

### Linking genetic ancestry to morphological traits

Comparison of mixed models with and without sex as a random effect showed lower AICc values for models excluding sex in all cases, except for CO (Table 2; Supplementary Data Fig. S6). Therefore, all reported models are linear models without sex except for those for CO (Table 2).

**Table 2.** Coefficients and significance levels of univariate and multivariate models between morphological traits (CA, calyx length; CO, corolla length; D_INFL, diameter of the inflorescence; HAIR, length of the longest calyx hair; LTOOTH, length of the longest calyx tooth; and PROP_LTOOTH, relative size of the longest calyx tooth compared with the calyx length) and percentage of genetic ancestry (groups 1–5) for individuals in each of the five genetic groups (Algarve, Cádiz, Doñana, Hercynian and Tetraploid; columns). All models correspond to linear models, except for those on CO, which were fitted using linear mixed models accounting for the sex of the individual as a random effect. Coefficients (Est.) and *P*-values (*P*) that are under the significance level (*P* < 0.05) are indicated in bold. In multivariate models, the ancestry corresponding to the group that is analysed are kept out of the models (cells in blank).

		Univariate models										Multivariate models									
		Algarve (*n* = 23)		Cádiz (*n* = 21)		Doñana (*n* = 67)		Hercynian (*n* = 8)		Tetraploid (*n* = 55)		Algarve (*n* = 23)		Cádiz (*n* = 21)		Doñana (*n* = 67)		Hercynian (*n* = 8)		Tetraploid (*n* = 55)	
	Predictors	Est.	*P*	Est.	*P*	Est.	*P*	Est.	*P*	Est.	*P*	Est.	*P*	Est.	*P*	Est.	*P*	Est.	*P*	Est.	*P*
CA	(Intercept)											**3**.**09**	**<0**.**001**	**2**.**83**	**<0**.**001**	**3**.**58**	**<0**.**001**	**5**.**55**	**<0**.**001**	**4**.**96**	**<0**.**001**
	group1 (Tetraploid)	−0.01	0.398	−0.02	0.063	0.02	0.369	**−0**.**03**	**0**.**026**	0.02	0.117	−0.00	0.685	−0.02	0.159	−0.00	0.976	−0.02	0.387		
	group2 (Hercynian)	0.06	0.085	0.01	0.821	**0**.**19**	**<0**.**001**	**0**.**02**	**0**.**007**	−0.04	0.069	0.06	0.188	−0.01	0.819	**0**.**13**	**<0**.**001**			−0.04	0.087
	group3 (Algarve)	0.01	0.611	0.13	0.108	**0**.**11**	**<0**.**001**	−0.04	0.082	0.01	0.761			0.09	0.227	**0**.**06**	**0**.**001**	−0.01	0.495	0.01	0.766
	group4 (Doñana)	−0.02	0.823	0.02	0.082	**−0**.**04**	**<0**.**001**	0.02	0.626	0.00	0.977	−0.00	0.992	0.01	0.274			0.00	0.996	−0.01	0.880
	group5 (Cádiz)	−0.06	0.632	−0.00	0.748	**−0**.**08**	**0**.**028**	**−0**.**09**	**0**.**012**	−0.04	0.342	−0.02	0.870			−0.03	0.192	−0.06	0.230	−0.03	0.452
CO	(Intercept)											**3**.**46**	**<0**.**001**	**2**.**82**	**<0**.**001**	**3**.**36**	**<0**.**001**	**3**.**70**	**<0**.**001**	**4**.**44**	**<0**.**001**
	group1 (Tetraploid)	−0.01	0.240	−0.01	0.746	0.00	0.922	0.03	0.090	0.01	0.654	−0.01	0.090	0.00	1.000	−0.00	0.561	**0**.**04**	**0**.**002**		
	group2 (Hercynian)	0.02	0.607	−0.04	0.449	**0**.**10**	**<0**.**001**	−0.01	0.192	−0.03	0.328	0.01	0.765	−0.06	0.141	**0**.**08**	**<0**.**001**			−0.04	0.151
	group3 (Algarve)	0.01	0.280	0.16	0.088	**0**.**06**	**<0**.**001**	0.03	0.238	0.03	0.528			0.09	0.194	**0**.**03**	**0**.**015**	0.02	0.187	0.05	0.288
	group4 (Doñana)	−0.04	0.558	**0**.**03**	**0**.**026**	**−0**.**02**	**0**.**002**	−0.04	0.414	0.01	0.794	−0.01	0.856	**0**.**03**	**0**.**003**			0.01	0.793	0.01	0.765
	group5 (Cádiz)	0.14	0.201	−0.02	0.109	−0.04	0.166	0.01	0.801	−0.01	0.840	0.05	0.524			0.01	0.694	**−0**.**07**	**0**.**009**	−0.00	0.956
D_INFL	(Intercept)											**8**.**27**	**<0**.**001**	**7**.**48**	**<0**.**001**	**9**.**58**	**<0**.**001**	**12**.**91**	**0**.**002**	**13**.**97**	**<0**.**001**
	group1 (Tetraploid)	0.01	0.467	0.01	0.859	0.01	0.772	−0.03	0.581	0.05	0.298	0.02	0.273	0.01	0.746	−0.01	0.676	−0.03	0.777		
	group2 (Hercynian)	0.11	0.067	0.07	0.569	**0**.**30**	**<0**.**001**	0.01	0.645	−0.07	0.237	0.11	0.083	0.06	0.669	**0**.**22**	**0**.**003**			−0.07	0.297
	group3 (Algarve)	−0.01	0.388	−0.08	0.676	**0**.**16**	**<0**.**001**	−0.03	0.687	−0.01	0.913			−0.10	0.649	0.09	0.073	−0.01	0.901	−0.03	0.842
	group4 (Doñana)	**−0**.**26**	**0**.**021**	0.01	0.603	**−0**.**06**	**0**.**002**	0.04	0.738	0.06	0.537	−0.21	0.051	0.02	0.592			0.02	0.901	0.05	0.646
	group5 (Cádiz)	−0.19	0.330	−0.01	0.530	−0.11	0.157	−0.03	0.826	−0.13	0.272	−0.05	0.780			−0.03	0.653	0.03	0.890	−0.12	0.346
HAIR	(Intercept)											**0**.**31**	**<0**.**001**	**0**.**28**	**<0**.**001**	**0**.**41**	**<0**.**001**	**0**.**88**	**0**.**001**	**0**.**74**	**<0**.**001**
	group1 (Tetraploid)	−0.00	0.340	−0.00	0.315	0.00	0.378	−0.00	0.774	0.00	0.421	−0.00	0.885	−0.00	0.964	0.00	0.471	−0.01	0.259		
	group2 (Hercynian)	**0**.**01**	**0**.**001**	0.00	0.827	**0**.**01**	**0**.**047**	−0.01	0.319	−0.00	0.398	**0**.**01**	**0**.**006**	−0.00	0.454	0.01	0.125			−0.00	0.408
	group3 (Algarve)	0.00	0.729	**0**.**04**	**0**.**004**	0.00	0.156	0.00	0.747	0.00	0.697			**0**.**03**	**0**.**002**	0.00	0.916	0.00	0.586	0.00	0.684
	group4 (Doñana)	−0.00	0.730	**0**.**01**	**<0**.**001**	−0.00	0.079	0.00	0.217	−0.00	0.614	0.00	0.859	**0**.**01**	**<0**.**001**			−0.00	0.978	−0.00	0.586
	group5 (Cádiz)	−0.01	0.289	**−0**.**00**	**0**.**011**	−0.00	0.878	−0.01	0.073	−0.00	0.772	−0.00	0.689			0.00	0.779	−0.00	0.907	−0.00	0.827
LTOOTH	(Intercept)											**1**.**70**	**<0**.**001**	**1**.**58**	**<0**.**001**	**1**.**97**	**<0**.**001**	**3**.**69**	**<0**.**001**	**3**.**24**	**<0**.**001**
	group1 (Tetraploid)	−0.00	0.485	−0.01	0.182	0.01	0.428	−0.02	0.065	0.01	0.224	−0.00	0.755	−0.01	0.350	−0.00	0.758	−0.01	0.679		
	group2 (Hercynian)	0.04	0.103	0.00	0.982	**0**.**16**	**<0**.**001**	**0**.**02**	**0**.**026**	−0.02	0.152	0.04	0.218	−0.01	0.755	**0**.**11**	**<0**.**001**			−0.02	0.195
	group3 (Algarve)	0.00	0.704	**0**.**10**	**0**.**041**	**0**.**09**	**<0**.**001**	−0.03	0.089	0.01	0.805			0.08	0.100	**0**.**05**	**<0**.**001**	−0.01	0.519	0.01	0.853
	group4 (Doñana)	−0.01	0.908	0.01	0.135	**−0**.**04**	**<0**.**001**	0.03	0.450	0.01	0.677	0.01	0.914	0.01	0.363			0.01	0.676	0.01	0.817
	group5 (Cádiz)	−0.06	0.503	−0.00	0.624	**−0**.**06**	**0**.**048**	**−0**.**07**	**0**.**020**	−0.04	0.233	−0.03	0.721			−0.02	0.340	−0.05	0.272	−0.03	0.322
PROP_LTOOTH	(Intercept)											**0**.**55**	**<0**.**001**	**0**.**56**	**<0**.**001**	**0**.**56**	**<0**.**001**	**0**.**66**	**<0**.**001**	**0**.**65**	**<0**.**001**
	group1 (Tetraploid)	0.00	0.961	0.00	0.605	0.00	0.796	−0.00	0.855	−0.00	0.567	0.00	0.854	0.00	0.599	−0.00	0.309	0.00	0.483		
	group2 (Hercynian)	0.00	0.479	−0.00	0.753	**0**.**01**	**<0**.**001**	0.00	0.686	0.00	0.634	0.00	0.553	−0.00	0.883	**0**.**01**	**<0**.**001**			0.00	0.516
	group3 (Algarve)	−0.00	0.832	0.01	0.283	**0**.**00**	**<0**.**001**	−0.00	0.385	0.00	0.922			0.01	0.288	**0**.**00**	**0**.**001**	−0.00	0.637	−0.00	0.934
	group4 (Doñana)	0.00	0.881	−0.00	0.902	**−0**.**00**	**<0**.**001**	0.00	0.230	0.00	0.160	0.00	0.768	−0.00	0.870			0.00	0.358	0.00	0.197
	group5 (Cádiz)	−0.01	0.521	−0.00	0.733	−0.00	0.387	−0.00	0.383	−0.00	0.234	−0.01	0.676			0.00	0.622	−0.00	0.489	−0.00	0.276

The models indicated pronounced variation among genetic groups in the influence of genetic ancestry on floral morphological traits. Overall, Doñana individuals exhibited the strongest and most consistent influence of genetic ancestry on floral morphology. In this group, ancestry from other diploid groups was significantly associated with most major traits examined (Table 2), with particularly positive contributions from Hercynian and Algarve lineages. These effects remained largely significant in multivariate models that included all ancestry groups, highlighting the robust and independent effect of genetic background.

In contrast, Tetraploid individuals exhibited non-significant associations in all cases (any combination of morphological trait and ancestry group). This lack of effects is evident in both univariate and multivariate models (Table 2). The remaining genetic groups displayed only sporadic, trait-specific associations, with most effects losing significance when changing from univariate to multivariate models (e.g. the negative effect of Tetraploid ancestry on CA in Hercynian individuals). The only effects that remained significant in both univariate and multivariate models were: (1) a negative effect of percentage of Hercynian ancestry on HAIR for Algarve individuals; (2) the positive effect of percentage of Doñana ancestry on CO and HAIR for Cádiz individuals; and (3) the positive effect of percentage of Algarve ancestry on HAIR for Cádiz individuals. Only two effects were significant in multivariate models but not in univariate models: (1) a negative influence of Cádiz ancestry; and (2) a positive influence of Tetraploid ancestry on CO in Hercynian populations. This was the only effect of Tetraploid ancestry detected in any diploid population, and although statistically significant, the estimated effect size was very small, indicating a weak signal.

*Ad hoc* analyses, in which we re-ran the morphological comparisons after excluding individuals from populations with mean self-ancestry values of <75 % (Fig. 2), revealed a much clearer differentiation between the Hercynian group and the other diploid groups. For example, calyxes of individuals within Hercynian group usually exceeded 5 mm, whereas the other diploid groups generally measured less. The Doñana group displayed significantly larger values of floral traits than those from Algarve and Cádiz, but significantly lower than Hercynian and the Tetraploid groups, although with some degree of overlap.

## DISCUSSION

Our results reveal a remarkable level of intraspecific complexity within *Thymus* sect. *Mastichina*, characterized by substantial overlap among genetic lineages at the morphological and ecological levels. Although some degree of differentiation was observed between diploid and tetraploid individuals, the high variability complicates taxonomic delimitation based on discrete morphological traits. This discordance between phenotypic variation and genetic structure is likely to reflect a combination of phenotypic plasticity, recent divergence or incomplete reproductive isolation (Villellas *et al.*, 2014; Castillo *et al.*, 2018). Taken together, these findings suggest that *Thymus* sect. *Mastichina* constitutes a complex of cryptic lineages, in which genetic differentiation is not consistently mirrored by phenotypic or ecological distinctiveness.

### Morphological variability

The morphological characterization of *Thymus* sect. *Mastichina* reveals significant differences primarily associated with ploidy level, whereas sexual dimorphism associated with the gynodioecious system plays a minor role in shaping floral morphology, with corolla size (CO) as the main exception (Delph *et al.*, 1996; Thompson *et al.*, 2002; Stakelienė and Ložienė, 2014). As expected, tetraploid individuals exhibit, on average, larger values of floral traits than their diploid counterparts, a pattern commonly attributed to polyploidy-induced increases in cell size and, consequently, in the size of reproductive organs (Levin, 1983; Ramsey and Ramsey, 2014). However, this trend is not consistent across all diploid populations. Individuals from the Hercynian group and from some populations within the Doñana genetic group consistently exhibited larger values of floral traits, comparable to those observed in tetraploid populations, in contrast to the smaller floral values observed in other southwestern diploid populations, particularly those within the Cádiz and Algarve genetic groups. Notably, tetraploid populations encompassed a wide range of flower sizes, including individuals overlapping with the smallest flowers observed among diploids. Similar patterns of morphological–genetic decoupling have been reported in other Mediterranean *Thymus* taxa (e.g. *Thymus herba-barona*; Molins *et al.*, 2011) and in other plant groups. In *Campanula*, for example, distinct polyploid lineages cannot be separated reliably based on morphological traits despite strong genomic differentiation (Crowl *et al.*, 2017; Crowl and Cellinese, 2017). In *Knautia*, different cytotypes and genome sizes show little correspondence with morphology, resulting in overlapping phenotypes among genetically distinct lineages (Frajman *et al.*, 2015). The overlapping ranges of floral size between certain tetraploid and diploid individuals in *Thymus* sect. *Mastichina* suggest that, although ploidy level can influence floral size (Landis *et al.*, 2020; Vilcherrez-Atoche *et al.*, 2022; García-Muñoz *et al.*, 2023), a complex interplay of genetic (Weiss *et al.*, 2005; Krizek and Anderson, 2013), ecological (Chen *et al.*, 2025; Day Briggs and Anderson, 2025) and ontogenetic (Diggle, 1997; Bull-Hereñu and Ronse De Craene, 2020) factors might also shape trait expression.

Although we hypothesized that interploidy genetic introgression could be influencing observed morphological traits, we found no clear evidence that introgression from tetraploids significantly affects the morphology of diploids, except for CO in Hercynian individuals. In contrast, introgression among diploid genetic groups seems to affect floral morphology significantly. Namely, ancestry from Hercynian and, to a lesser extent, from Algarve lineages seems to influence floral size in Doñana individuals, potentially contributing to the high phenotypic variability observed in this group. García-Cárdenas *et al.* (2026) postulated that the Doñana group is likely to be the most recently established, more probably from the adjacent groups of Cádiz or Algarve. Nevertheless, admixture with the Hercynian group appears to be responsible for the longer morphological traits, particularly CA and LTOOTH, observed in this group, which might explain part of the observed morphological overlap.

### Ecological niches

The analysis of environmental conditions revealed significant differences between ploidy levels, with the tetraploid cluster occupying a distinct ecological niche relative to the diploid groups (with some exceptions; see below). These findings suggest niche differentiation associated primarily with ploidy level rather than among diploid lineages, consistent with previous studies highlighting the ecological significance of polyploidy (e.g. Ramsey, 2011; Soltis *et al.*, 2014). However, the diploid Hercynian group partly overlaps with the Tetraploid group in both geographical distribution and environmental conditions. In this context, García-Cárdenas *et al.* (2026) suggested that the Hercynian diploids might represent a relict lineage derived from an ancestral diploid population largely replaced by tetraploids throughout much of its range. Meanwhile, the southwestern Iberian Peninsula might have functioned as a Pleistocene glacial refugium, offering long-term environmental stability that facilitated the persistence and divergence of isolated lineages, promoting speciation and the retention of distinctive genetic and morphological traits in diploid taxa from this region (Girón *et al.*, 2012).

### Taxonomic and conservation implications

The limited morphological resolution observed across populations of this section underscores a broader challenge in plant systematics: the frequent mismatch between phenotype and underlying genetic structure, especially in recently diverged or rapidly evolving groups (Bickford *et al.*, 2007; Struck *et al.*, 2018). As humans, we depend strongly on visual information to interpret the natural world, and classical approaches to species classification have therefore been based mainly on morphological traits (Bickford *et al.*, 2007; Hending, 2025). However, in taxonomically complex groups, morphological traits alone are often insufficient for species delimitation. Addressing this issue ideally requires the integration of multiple lines of evidence to clarify species boundaries and support appropriate conservation actions (Federici *et al.*, 2013; Fišer *et al.*, 2018). Molecular approaches, such as DNA barcoding, have proved effective for species identification (Antil *et al.*, 2023) and are increasingly applied to detect cryptic species (e.g. Nieto-Lugilde *et al.*, 2024). Genome size has also emerged as a reliable character for species delimitation (Kron *et al.*, 2007; Fomicheva and Domblides, 2023). Additionally, integrative taxonomy considers other sources of information, such as geographical distribution and niche differentiation, further strengthening species delimitation (Dayrat, 2005; De Luna-Bonilla *et al.*, 2024; Wang *et al.*, 2025). Previous genomic work in *Thymus* sect. *Mastichina* (García-Cárdenas *et al.*, 2026) provides a useful foundation for developing DNA barcoding tools, although additional research is still required. In our study system, ploidy level constitutes a more reliable diagnostic trait than flower morphology, and we therefore advocate its incorporation into taxonomic assessments. Flow cytometry is widely used for estimating plant genome sizes and is an accessible method that can be conducted in external specialized laboratories at relatively low cost (Galbraith *et al.*, 2021; Sliwinska *et al.*, 2022). Thus, following Girón *et al.* (2012) and García-Cárdenas *et al.* (2026), we can establish a reliable distinction between species within *Thymus* sect. *Mastichina*: diploid individuals with 2C DNA content from 1.32 to 1.84 pg correspond to *T. albicans* Hoffmanns. & Link, whereas tetraploids with 2C DNA values from 2.31 to 3.07 pg should be considered as *T. mastichina* L.

Although we detected a considerable overlap in ecological niches, this pattern was mainly driven by subcoastal diploid populations (Algarve, Doñana and Cádiz groups). In contrast, the diploid Hercynian group occupies a distinctive ecological niche more closely resembling that of the Tetraploid group and is also morphologically and genetically distinct from other diploid lineages (García-Cárdenas *et al.*, 2026). Taken together, these ecological, morphological and genomic differences support its recognition as a distinct evolutionary lineage from *T. albicans* that might warrant species status. Despite being cryptic with respect to *T. mastichina* in floral morphology, ecological niche and geographical distribution, it can be distinguished reliably based on its genome size. Given that this lineage is currently known from only two populations, we adopt a cautious approach and defer formal taxonomic recognition to future work.

Regarding the Doñana genetic group, the high variability in floral traits can be explained, in part, by introgression, primarily with the Hercynian group. In fact, introgressed populations of the Doñana group exhibited the largest calyx size and were previously identified as *Thymus mastichina* subsp. *mastichina* (Girón *et al.*, 2012). Given that the Hercynian group is cryptic with *T. mastichina*, it is unsurprising that these populations were previously assigned to that taxon. If the Hercynian lineage is formally recognized as a species, these populations could perhaps be considered of hybrid origin, although they remain cryptic relative to *T. mastichina*.

After excluding these introgressed populations, Doñana individuals display intermediate floral trait values, larger than those from Algarve and Cádiz groups but smaller than those of Hercynian and Tetraploid genetic groups. Most of these individuals were formerly recognized as *T. mastichina* subsp. *donyanae*, using a 4 mm calyx-length threshold in identification keys (Morales, 2010), which corresponds to the mean value found for the Doñana group in our analyses **(**Supplementary Data Fig. S7). However, some overlap persists, particularly with smaller-flowered of Cádiz and Algarve populations. This complicates identification of new discovered populations, a matter of particular relevance for conservation purposes (L. Fernández-Carrillo, pers. comm.). Given that genomic data place the Doñana group within *T. albicans* and given the geographical structuration of the lineages (García-Cárdenas *et al.*, 2026), we propose a pragmatic subspecific framework combining morphological and geographical criteria to capture intraspecific diversity. Under this framework, diploid populations in SW Iberia with calyces ∼4 mm located between the Guadiana and Guadalquivir rivers would be assigned to the combination *Thymus albicans* subsp. *donyanae* (R.Morales) Rivas Mart. (Rivas Martínez, 1988). Populations located east of the Guadalquivir River and west of the Guadiana River, despite their disjunct distribution, could be referred to *T. albicans* subsp. *albicans*, based on their morphological (calyces ∼3 mm long) and ecological similarities.

The recognition of the Doñana populations as *Thymus albicans* subsp. *donyanae* would have relevant implications for conservation, because *T. albicans* is an endangered species (Moreno, 2008; Buira *et al.*, 2017). Following IUCN criteria (IUCN, 2012) and given the geographically intermediate position of Doñana populations between the Cádiz and Algarve populations (the sole formerly recognized as *T. albicans*) the extent of occurrence would remain geographically restricted to SW Iberia and comparable to previous estimates. However, the area of occupancy would increase owing to the inclusion of additional populations. The main threats affecting *T. albicans*, land-use change particularly associated with coastal urban pressure and greenhouse agriculture, are also present across the range of *T. albicans* subsp. *donyanae*, although a proportion of its populations occur within protected areas. A comprehensive conservation assessment is beyond the scope of this study and should be addressed once the taxonomic framework is resolved. Moreover, given the influence of introgression on flower morphology, we would recommend managing some Doñana populations as differentiated conservation units to safeguard the genetic and morphological integrity of other diploid groups, particularly the morphologically homogeneous and genetically distinct Cádiz group. Future work must define the relevant genetic units, evaluate current and emerging threats across populations, and consider the evolutionary potential of these distinct groups to inform effective long-term conservation strategies.

### Conclusion

Our integrative analysis indicates that *Thymus* sect. *Mastichina* represents a complex of cryptic evolutionary lineages characterized by extensive morphological overlap and with limited ecological differentiation among diploid groups, despite a marked genetic structuring. Although ploidy level influences floral traits and ecological niches, phenotypic variation is often decoupled from genetic lineages, possibly owing to recent divergence and introgression. Contrary to our expectations, introgression among diploid lineages (rather than interploidy introgression) appears to affect floral morphology, particularly contributing to phenotypic overlap in some lineages. These results highlight the limitations of morphology-based taxonomy in this group and emphasise the need for an integrative framework, proposing the use not only of morphology, but also of genome size and geographical distribution for species delimitation.

## Supplementary Material

mcag151_Supplementary_Data

## Data Availability

The data and code pertaining to the implemented analyses through Zenodo repository (https://doi.org/10.5281/zenodo.17938366).

## SUPPLEMENTARY DATA

Supplementary data are available at *Annals of Botany* online and consist of the following. Figure S1: comparison of morphological traits between ploidy levels and genetic groups for microscopic variables (stoma and pollen grain sizes). Figure S2: principal components analysis (PCA) plots depicting variation between diploid (orange squares) and tetraploid (blue circles) individuals based on measurements of the following six floral traits. Figure S3: principal components analysis (PCA) plots depicting variation among the five genetic clusters described by García-Cárdenas *et al.* (2026). Figure S4: principal component analysis (PCA) of soil variables for the samples collected at populations of *Thymus* sect. *Mastichina* across ploidy levels and genetic groups. Figure S5: multivariate patterns of climate characteristics based on a principal component analysis (PCA). Figure S6: effect of sex in floral morphological traits across ploidy levels and specifically in corolla length across ploidy levels and genetic groups. Table S1: populations of *Thymus* sect. *Mastichina*. Table S2: descriptive statistics at the ploidy level and genetic groups for morphological traits measured individuals of *Thymus* sect. *Mastichina*. Table S3: descriptive statistics at the population level for morphological traits measured individuals of *Thymus* sect. *Mastichina*. Table S4: comparison of alternative taxonomic grouping hypotheses based on morphological traits using model-based discriminant analysis (MclustDA). Table S5: pairwise directional niche overlap among the five genetic groups estimated using Bayesian niche region analyses implemented in the *nicheROVER* R package.
